# Functional Characterization of AP2/ERF Transcription Factors during Flower Development and Anthocyanin Biosynthesis Related Candidate Genes in *Lycoris*

**DOI:** 10.3390/ijms241914464

**Published:** 2023-09-23

**Authors:** Zhong Wang, Guowei Song, Fengjiao Zhang, Xiaochun Shu, Ning Wang

**Affiliations:** 1 Institute of Botany, Jiangsu Province and Chinese Academy of Sciences (Nanjing Botanical Garden Memorial Sun Yat-Sen), Nanjing 210014, China; wangzhong@cnbg.net (Z.W.); sgw12311@163.com (G.S.); fengjiao@cnbg.net (F.Z.); sxc@cnbg.net (X.S.); 2 Jiangsu Key Laboratory for the Research and Utilization of Plant Resources, Jiangsu Provincial Platform for Conservation and Utilization of Agricultural Germplasm, Nanjing 210014, China; 3 National Key Laboratory of Crop Genetics and Germplasm Enhancement, National Center for Soybean Improvement, Key Laboratory for Biology and Genetic Improvement of Soybeans (General, Ministry of Agriculture), Jiangsu Collaborative Innovation Center for Modern Crop Production, Nanjing Agricultural University, Nanjing 210095, China

**Keywords:** *Lycoris*, AP2/ERF transcription factors, expression patterns, flower development, anthocyanin biosynthesis, regulatory networks

## Abstract

The APETALA2/ethylene-responsive transcription factor (AP2/ERF) family has been extensively investigated because of its significant involvement in plant development, growth, fruit ripening, metabolism, and plant stress responses. To date, there has been little investigation into how the *AP2/ERF* genes influence flower formation and anthocyanin biosynthesis in *Lycoris*. Herein, 80 putative *LrAP2/ERF* transcription factors (TFs) with complete open reading frames (ORFs) were retrieved from the *Lycoris* transcriptome sequence data, which could be divided into five subfamilies dependent on their complete protein sequences. Furthermore, our findings demonstrated that genes belonging to the same subfamily had structural similarities and conserved motifs. *LrAP2/ERF* genes were analyzed for playing an important role in plant growth, water deprivation, and flower formation by means of gene ontology (GO) enrichment analysis. The expression pattern of the *LrAP2/ERF* genes differed across tissues and might be important for *Lycoris* growth and flower development. In response to methyl jasmonate (MeJA) exposure and drought stress, the expression of each *LrAP2/ERF* gene varied across tissues and time. Moreover, a total of 20 anthocyanin components were characterized using ultra-performance liquid chromatography-electrospray ionization tandem mass spectrometry (UPLC-ESI-MS/MS) analysis, and pelargonidin-3-O-glucoside-5-O-arabinoside was identified as the major anthocyanin aglycone responsible for the coloration of the red petals in *Lycoris*. In addition, we mapped the relationships between genes and metabolites and found that *LrAP2/ERF16* is strongly linked to pelargonidin accumulation in *Lycoris* petals. These findings provide the basic conceptual groundwork for future research into the molecular underpinnings and regulation mechanisms of AP2/ERF TFs in anthocyanin accumulation and *Lycoris* floral development.

## 1. Introduction

The AP2/ERF (APETALA 2/Ethylene responsive element binding factor) family is one of the most abundant transcription factors (TFs) in many different plants [1]. The AP2/ERF TF encodes a 60–70 amino acid protein, primarily containing a binding site for DNA, a domain engaged in oligomerization in the activation or suppression of transcription, a motif for, and a signal for nuclear localization [2]. Five distinct subfamilies of AP2/ERF TFs have been identified depending on the number and type of certain conserved AP2 motif and B3 motif: AP2 (APETALA2), DREB (dehydration responsive element-binding), ERF (ethylene responsive element-binding protein), RAV (related to ABI3/VP), and soloist [2]. Genes belonging to the AP2 subfamily contain two AP2 domains, which are involved in the development of flowers and seeds [3,4]. There is just one AP2 domain shared by the ERF and DREB subfamilies. This domain is the largest gene number in the AP2/ERF family and performs critical functions in plant defense against both biotic and abiotic stressors [5]. Genes belonging to the ERF subfamily exert critical functions in the plant’s responses to biotic stress and pathogens [6]. The genes that belong to the DREB subfamily are the primary modulators of abiotic stress in plants and are prominent under cold and osmotic stress [7]. A single AP2 domain and a single B3 domain make up the RAV subfamily, which functions in floral development, bud expansion, leaf senescence, and defense against pathogens and environmental stressors [8]. Soloist is a small subfamily of genes possessing a single AP2 domain, but their sequences are highly divergent from those of other *AP2/ERF* genes [9]. These genes contribute to defense responses against salt stress and pathogens [10,11]. Numerous *AP2/ERF* genes have been extracted from a broad variety of plant species, and they are now often used in efforts to increase crop resilience to stress [12,13]. DREB family members are capable of precisely binding to DRE/CRT *cis*-acting regions, which allows them to regulate responses to ABA, low temperatures, and drought, whereas the GCC-box, which is involved in ethylene response, resistance to diseases, and abiotic stress, is the preferred binding site for the ERF subfamily members [14]. *AP2/ERF* transcription factors have been found throughout the genomes of various model plants such as *Arabidopsis thaliana* and *Oryza sativa* [2,15] and in other plants like *Cajanus cajan*, *Cicer arietinum*, *Camellia sinensis*, *Dimocarpus longan*, *Glycine max*, *Panax ginseng*, *Pyrus pyrifolia*, *Salix matsudana*, *Rhododendron simsii*, *Triticum aestivum*, *Zea mays,* and *Zingiber officinale* [4,16,17,18,19,20,21,22,23,24,25]. Nevertheless, these findings are based on a very limited number of the thousands of plant species. Gene mining for *AP2/ERF* in *Lycoris* plants has so far been relatively spotty and inconclusive. Therefore, additional studies on *Lycoris* plants as well as identification methods that are more accurate and comprehensive are necessary.

In previous research, *AP2/ERF* genes were shown to have a strong link to the flowering process, particularly genes belonging to the AP2 family [26,27]. Flowering is generally acknowledged as an important signal in the plant’s shift from vegetative development to the reproductive phase [28]. Illustrating the mechanisms of flower development and flower transition has great significance for plant adaptation to poor environments and plant breeding [29]. There is plenty of evidence of the implication of *AP2/ERF* genes in flower development processes. For instance, in the ‘ABC’ model of flower development, APETALA2 (AP2), an A-class gene, took part in flower formation [30]. Recent scientific investigations have illustrated that the levels of two *DoAP2* genes (*DoAP2-8* and *DoAP2-10*) are lowered during the flowering process, whereas the levels of two *AP2* genes (*DoAP2-2* and *DoAP2-3*) are elevated, indicating that *DoAP2* genes play a role in both plant regeneration and flower development in *Dendrobium officinale* [31]. The upregulation of miR172, which targeted the AP2-like gene, caused roses to develop a trait known as double flowers [32]. Furthermore, the AP2-like TF known as *TOE1* has the potential to modulate FT expression, which in turn controls flowering [33]. In flowering plants, *SOC1* could cooperate with the *CBF* promoter to affect flowering in a cold environment [34].

As per the findings of multiple studies, interactions between the *AP2/ERFs* and MBW complex are shown to have a role in the regulation of anthocyanin biosynthesis, and they exhibit a variety of control mechanisms for anthocyanin accumulation, which vary depending on the species of plant and the environmental circumstances [35,36,37,38,39,40,41]. In phytohormone-induced anthocyanin biosynthesis, *MdERF1B* and *MdERF3* directly activate the expression of *MdMYB11* and *MdMYB1*, respectively, thereby promoting ethylene-induced anthocyanin biosynthesis in apple fruits [36,42]; jasmonate and ethylene-regulated *PyERF22* could enhance the activation of the *PyUFGT* promoter by interacting with *PyMYB10* and *PyMYB10b* to promote lanolin-induced anthocyanin biosynthesis in ‘Zaosu’ pear fruits (*Pyrus pyrifolia Nakai*) [37]; *PyERF105*, which is activated by ethylene, stimulates the expression of the repressive *PyMYB140*, thus inhibiting the synthesis of anthocyanin in red-skinned pear fruits [38]. *Pp4ERF24* and *Pp12ERF96* were found to interface with *PpMYB114* to enhance the crosstalk between *PpMYB114* and *PpAP2/ERF3* in anthocyanin accumulation in response to light in red pear (*Pyrus pyrifolia*) [43]. *MdERF38* interacts with *MdMYB1* for the purpose of promoting drought stress-induced anthocyanin biosynthesis in apple fruits [44]. In addition, the MdERF109 protein is responsible for the early induction of anthocyanin-associated gene expression as well as the accumulation of anthocyanins throughout the coloring process of apples [45]. *ERF5* can control the anthocyanin accumulation process through its interactions with *MYBA* and *F3H* genes in ‘Zijin’ mulberry fruits [39]. Thus, the above studies showed that many *AP2/ERFs* positively regulate anthocyanin biosynthesis, whereas a negative regulation of anthocyanin biosynthesis by repressor-type *AP2/ERFs* in horticultural plants is unclear. Meanwhile, the color of red-skinned pears is often unstable and sometimes does not develop, which seriously restricts the development of the red-skinned pear industry [38]. *PpERF9* represses *PpRAP2.4* and *PpMYB114* via histone deacetylation to inhibit anthocyanin biosynthesis in pear [40]. *PyERF4.1/PyERF4.2* interacted with *PyERF3* to affect the stability of the *PyERF3-PyMYB114-PyAP2/ERF3* complex and inhibited anthocyanin biosynthesis in red-skinned pears [41]. Therefore, it is of great significance and necessity to explore how *AP2/ERFs* promote anthocyanin biosynthesis and thus balance the abnormal accumulation of anthocyanins in *Lycoris* flowers.

*Lycoris* is subjected to various environmental conditions, including drought and high temperatures, which both retard the plant rate of development and reduce its overall output [46,47]. Through transcriptome sequencing initiatives, a large number of genes in *Lycoris* potentially involved in the biosynthesis of alkaloids, flavonoids, and polysaccharides have been found [48,49,50]. In *Lycoris sprengeri*, 6-benzyladenine application did not affect the biological processes among groups but caused a premature decrease in sucrose and total soluble sugar content. Moreover, a relatively active cell wall invertase-catalyzed pathway can increase bulblet regeneration, affecting bulb yield at the competence stage [49]. Upstream pathways for strictosidine biosynthesis in plants have been preserved and well-defined. Transcription factors (TF) are important gene expression switches that activate or repress the expression of specific target genes by interacting with *cis*-elements in the gene promoter region, regulating various biological processes including plant development, growth, biosynthesis of secondary metabolites, and response to stresses [5,6,7]. The *AP2/ERF* TFs present in *Lycoris* and their expression patterns during *Lycoris* flower development have remained largely unknown. *Lycoris longituba* and *Lycoris radiata* transcriptome sequence data [50,51] provide an important resource for a comprehensive study of the *Lycoris AP2/ERF* family by using a bioinformatics approach. Furthermore, we studied the expression patterns of *AP2/ERF* genes in distinct tissues, flowering stages of *Lycoris*, as well as various time points following MeJA treatment and exposure to drought stress. By integrating gene expression and anthocyanin metabolome analysis, we detected *LrAP2/ERFs*, the essential structural genes, and metabolites that are involved in anthocyanin accumulation, to help explain the molecular process of petal pigment generation. Additionally, the probable protein–protein interactions (PPI) of *LrAP2/ERFs* were also predicted. Our research offered critical knowledge for evaluating key *AP2/ERF* genes in controlling flowering and anthocyanin accumulation in woody perennials and shed new light on the evolutionary changes of *AP2/ERF* genes in *Lycoris*.

## 2. Results

### 2.1. Identification, Physicochemical Properties, and Subcellular Localization Prediction of Lycoris AP2/ERF TFs

The prediction of *AP2/ERF* superfamily genes was vastly duplicated in *Lycoris*. We searched the *L. radiata* and *L. longituba* transcriptomic datasets for probable LrAP2/ERF protein sequences utilizing HMMER 3.0 and BLASTP with an E-value cutoff of <1 × 10^−5^. All candidate sequences were confirmed in NCBI and with SMART to further identify conserved complete AP2/ERF domains. Eighty LrAP2/ERF proteins were identified within the range of LrAP2/ERF1 to LrAP2/ERF80 (Table S1a). The physicochemical properties of the *LrAP2/ERF* TF family are depicted in Table S1a. The CDS lengths of the *LrAP2/ERF* genes were within the range of 378 bp to 1452 bp. The molecular weights of LrAP2/ERF proteins varied between 14.12 and 54.09 kDa, their isoelectric values were within the range of 4.93 to 10.35, and their lengths varied from 125 to 483 amino acids. The subcellular localization of the LrAP2/ERF proteins was predicted by using ProtComp 9.0, Plant-mPLoc, and WOLF PSORT. The majority of LrAP2/ERF proteins were presumably positioned in the nucleus, whereas only a small number of these proteins were found in the cytoplasm, chloroplasts, and mitochondria. It showed that 37 LrAP2/ERF TFs (or 46.25%) were localized in the nucleus; thirteen (16.25%) of the LrAP2/ERF TFs were found in the nucleus and chloroplasts; a total of 11 LrAP2/ERF TFs (13.75%) were found in both the cytoplasm and the nucleus; the chloroplast hosted eight LrAP2/ERF TFs (10.0%); and a total of four LrAP2/ERF TFs (5.0%) were found in both mitochondria and nucleus. Notably, chloroplast and mitochondrial localization were observed for LrAP2/ERF52 and LrAP2/ERF72, whereas cytoplasmic localization was shown for LrAP2/ERF19 (Table S1a). Nucleus-expressed LrAP2/ERF proteins predominated and were largely hydrophilic, acidic, and unstable.

### 2.2. Characterization and Analysis of the Phylogenetic Tree of LrAP2/ERF Proteins

**Figure 1:**
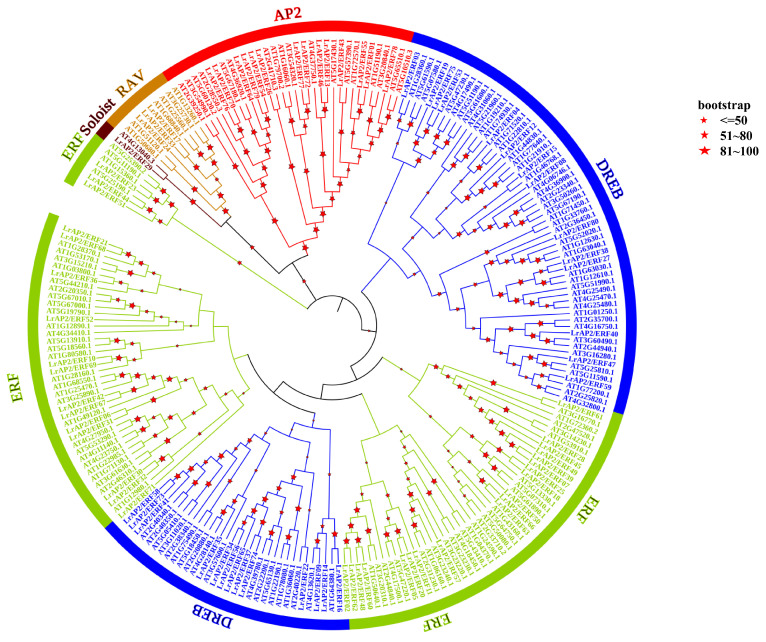
Phylogenetic analysis of LrAP2/ERFs in *Lycoris*. The phylogenetic tree is constructed via the NJ method on the basis of the alignment of AP2/ERF domains. Only the percentages of replicate trees greater than 50 are shown on the branches, and these values are calculated by bootstrap tests (1000 replicates) for reliability verification. The tree shows the five subfamilies highlighted in different colors.

To investigate the evolutionary relationships of LrAP2/ERF and AtAP2/ERF proteins, a phylogenetic tree was generated (Figure 1). It showed the tree topology and categorization of 80 LrAP2/ERF proteins were grouped into 5 subfamilies, including DREB, AP2, ERF, RAV, and soloist, mirroring the organization and categorization of AP2/ERF proteins in *A. thaliana*. The largest number of *LrAP2/ERF* genes were found in the ERF subfamily, followed by the DREB, AP2, and RAV subfamilies. The ERF subfamily in *Lycoris* was represented by 35 genes, whereas the DREB subfamily was found to be primarily composed of 27 genes. The AP2 subfamily accounted for around 17.5% of the *LrAP2/ERF* genes (14 genes). The RAV family is composed of three genes, each of which has a single AP2 domain and a single B3 domain. In addition, *LrAP2/ERF29* was identified as a soloist in *Lycoris* as they had high homology with *AT4G13040.3* in *A. thaliana*. These findings indicated that AP2/ERF proteins were present in a wide range of plant species before their diversification and that they subsequently underwent independent evolution within each species.

### 2.3. Conserved Motifs and AP2/ERF Domains of LrAP2/ERF Proteins

**Figure 2:**
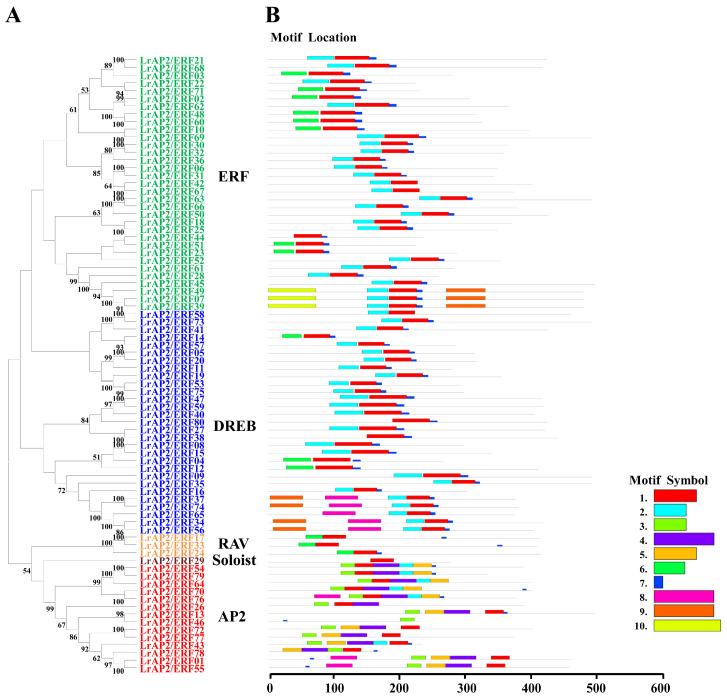
Phylogenetic relationships and conserved motif analysis of LrAP2/ERF proteins. (**A**) Neighbor-joining phylogenetic tree of LrAP2/ERF (bootstrap values for 1000 replicates). (**B**) Distribution of conserved motifs in LrAP2/ERF proteins. The colored boxes represent different motifs. The box length represents motif length.

The LrAP2/ERF protein sequences of *Lycoris* were aligned to discover more about the structural features of the genes in the LrAP2/ERF family. The conserved motif and domain of LrAP2/ERFs were further investigated using MEME (Figure 2 and Figure S1). Using the protein sequences of LrAP2/ERF, we predicted ten distinct motifs (Figure 2). We compiled a summary of the motif patterns found in each subfamily and discovered that all LrAP2/ERF subfamilies share motif 1 (Figure 2). The RAV subfamily contains motif 1, motif 3, and motif 7, whereas the DREB subfamily has motif 8 in addition to the motifs found in the AP2 subfamily. Motif 9 is unique to the DREB family and is found in a non-AP2 domain (Figure 2). Furthermore, four to eight motifs were found in each of the 14 AP2 subfamily genes. Motif 4, motif 5, and motif 6 are the most common and may be found in any gene belonging to the AP2 subfamily. These findings demonstrate the varying roles played by *LrAP2/ERF* members (Figure 2). These findings imply that variations in amino acid sequences provide many members of the LrAP2/ERF family with unique characteristics. These conserved motifs are involved in a variety of biochemical functions, such as transcriptional activity, PPIs, protein interaction, and the structural configuration of proteins.

### 2.4. Gene Ontology Annotation Enrichment between LrAP2/ERF Transcription Factor Genes

**Figure 3:**
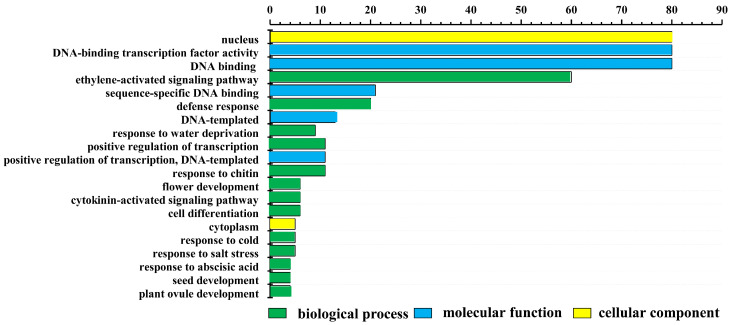
Gene ontology (GO) enrichment analysis of LrAP2/ERF proteins. The top 20 GO terms of level 2 in molecular function, biological process, and cellular component were visualized. The x-axis shows the enriched protein numbers, and the y-axis shows the information about GO terms.

To further characterize the functions of LrAP2/ERF transcription factors, all LrAP2/ERFs were subjected to GO annotation and enrichment analysis. The LrAP2/ERF transcription factors were classified into 93 different functional categories and grouped as per the following three ontologies: biological processes (BPs), cellular components (CCs), and molecular functions (MFs). At level two, the top 20 GO terms were ranked and visualized (Figure 3). In CC terms, the nucleus contained 80 of the LrAP2/ERF proteins, whereas the cytoplasm, cytosol, and plasma membrane only contained a few of these proteins. In MF terms, it was determined that 80 LrAP2/ERF proteins exhibited DNA-binding TF activity (Figure 3). In BP terms, the ethylene-activated signaling pathway was linked to 60 LrAP2/ERF proteins. Significant enrichment of target genes in GO terms was observed in response to abscisic acid, cold stress, salt stress, water deprivation, the cytokinin-activated signaling pathway, flower development, seed development, and plant ovule development.

### 2.5. Analysis of LrAP2/ERF Gene Expression Patterns in Various Tissues

**Figure 4:**
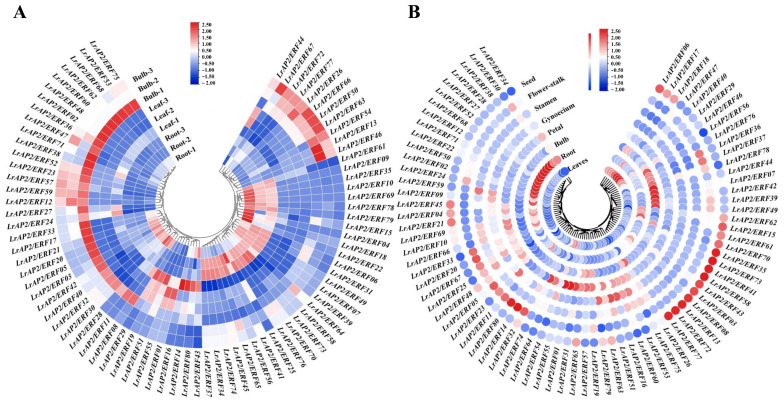
Expression analysis of *LrAP2/ERF* genes across different tissues. (**A**) Expression profile heatmap with hierarchal clustering of *LrAP2/ERFs* at different developmental stages of *Lycoris longituba*. Samples were obtained from the roots, leaves, and bulbs of 4-year-old plants. (**B**) *LrAP2/ERFs* gene expression profiles at different developmental stages of *L. radiata*. The relative expression for each gene is depicted by color intensity in each field. Higher values are represented by red, whereas lower values are represented by blue.

As the transcriptome analysis of different tissues (root, leaf, and bulb) has been revealed in *L. longituba* [51], we used local blast within *L. longituba* transcriptomic data to screen for the orthologous genes of *LrAP2/ERF*. Although the expression of certain *LrAP2/ERF* genes varied across the three tissues, the levels of other associated genes were consistent across tissues (Figure 4A). For instance, most *LrAP2/ERF* genes exhibited higher expression levels in the roots and bulbs than in the leaves. Five *LrAP2/ERFs* genes, including *LrAP2/ERF01*, *LrAP2/ERF19*, *LrAP2/ERF29*, *LrAP2/ERF51*, and *LrAP2/ERF55,* were highly expressed in leaves, whereas 12 *LrAP2/ERF* genes were preferentially expressed in roots. In addition, *LrAP2/ERF12*, *LrAP2/ERF23*, *LrAP2/ERF26*, *LrAP2/ERF50*, *LrAP2/ERF52*, *LrAP2/ERF57*, *LrAP2/ERF59*, *LrAP2/ERF63*, *LrAP2/ERF66*, *LrAP2/ERF72,* and *LrAP2/ERF77* were substantially highly expressed in bulbs. To further elucidate the biological function of LrAP2/ERF proteins, qRT-PCR was utilized to determine the spatial specificity of the expression pattern of 80 *LrAP2/ERF* genes in eight *L. radiata* organs. Figure 4B shows that *LrAP2/ERF* genes are functionally differentiated throughout plant growth, as certain *LrAP2/ERF* genes demonstrated differential expression in the eight *L. radiata* tissues while other *LrAP2/ERF* genes had identical expression profiles in varied tissues. For instance, four *LrAP2/ERFs* (i.e., *LrAP2/ERF29*, *LrAP2/ERF40*, *LrAP2/ERF46*, and *LrAP2/ERF47*) showed considerably high expression levels in leaves. *LrAP2/ERF01*, *LrAP2/ERF31*, *LrAP2/ERF55*, and *LrAP2/ERF74* were preferentially expressed in petals. *LrAP2/ERF16* and *LrAP2/ERF51* exhibited significant expression profiles in gynoecium, whereas *LrAP2/ERF59* showed substantial up-regulation in stamens. Also, five *LrAP2/ERFs* (i.e., *LrAP2/ERF07*, *LrAP2/ERF39*, *LrAP2/ERF42*, *LrAP2/ERF44*, and *LrAP2/ERF49*) were considerably increased in bulb tissues. Moreover, *LrAP2/ERF02*, *LrAP2/ERF09*, *LrAP2/ERF12*, *LrAP2/ERF71*, *LrAP2/ERF22*, *LrAP2/ERF24*, *LrAP2/ERF27*, *LrAP2/ERF28*, *LrAP2/ERF38*, *LrAP2/ERF50*, *LrAP2/ERF52*, and *LrAP2/ERF68* were predominantly expressed in roots. *LrAP2/ERF03*, *LrAP2/ERF08*, *LrAP2/ERF13*, *LrAP2/ERF35*, *LrAP2/ERF41*, *LrAP2/ERF43*, *LrAP2/ERF58*, *LrAP2/ERF72*, *LrAP2/ERF73*, and *LrAP2/ERF77* were relatively highly abundant in seeds. Among all the tissues, the fewest *LrAP2/ERF* members were expressed the most in the roots and flower stalk. Conversely, some genes were not expressed specifically. Based on these findings, it seems that *LrAP2/ERFs* may have a similar function in *Lycoris* growth and expansion. Furthermore, most *LrAP2/ERFs* showed highly tissue-specific expression profiles, which is suggestive of their wide range of roles in various organs.

### 2.6. Expression Patterns Analysis of LrAP2/ERF Genes during Lycoris Flower Developmental Stages

**Figure 5:**
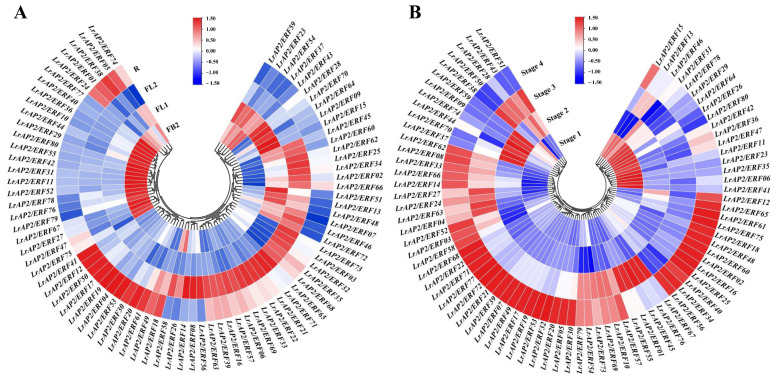
Expression profile with hierarchal clustering of *LrAP2/ERFs* during flower developmental stages. (**A**) Gene expression profiles of *LrAP2/ERFs* at different flower developmental stages of *L. radiata*. (**B**) Expression profile heatmap with hierarchal clustering of *LrAP2/ERFs* at different flower developmental stages of *L. longituba*. The relative expression for each gene is depicted by color intensity in each field. Higher values are represented by red, whereas lower values are represented by blue.

On the basis of tissue-specific expression, the expression of *LrAP2/ERF* was further observed at the flowering developmental stages using previously published RNA-seq data and the qRT-PCR technique (Figure 5). In *L. radiata* flowering development, FB (S1 stage), FL1 (S2 stage), FL2 (S3 stage), and R (S4 stage) indicated the initial stage of flower-bud differentiation, female flower primordium, early blooming stage, and full blooming stage, respectively (Figure 5A) [50]. More than half of the *LrAP2/ERF* genes have a low expression level at the S1 stage and are subsequently significantly elevated at the S4 stage. Among *LrAP2/ERFs*, 13 genes (*LrAP2/ERF02*, *LrAP2/ERF07*, *LrAP2/ERF13*, *LrAP2/ERF25*, *LrAP2/ERF34*, *LrAP2/ERF45*, *LrAP2/ERF46*, *LrAP2/ERF48*, *LrAP2/ERF51*, *LrAP2/ERF60*, *LrAP2/ERF62*, *LrAP2/ERF66*, and *LrAP2/ERF72*) showed rising expression tendencies as the flowers bloomed, notably in the transition from S1 to S3 stage. In contrast, 19 *LrAP2/ERF* genes were declined during flower development. Moreover, most genes showed persistent trends during floral development, and the expression of several genes peaked at stages 3 and 4. Figure 5B demonstrates that the *LrAP2/ERF* genes exhibited tissue-specific expression profiles, with lower levels in reproductive organs, after assessing the expression bias of *LrAP2/ERFs* throughout four distinct *L. longituba* flower development stages. In the flowering process, *LrAP2/ERF06*, *LrAP2/ERF11*, *LrAP2/ERF23*, *LrAP2/ERF26*, *LrAP2/ERF29*, *LrAP2/ERF35*, *LrAP2/ERF36*, *LrAP2/ERF41*, *LrAP2/ERF42*, *LrAP2/ERF47*, *LrAP2/ERF64*, *LrAP2/ERF78*, and *LrAP2/ERF80* showed high transcript levels in the S1 stage (Figure 5B). The analysis of the results showed that *LrAP2/ERF09*, *LrAP2/ERF44*, *LrAP2/ERF59*, *LrAP2/ERF70*, and *LrAP2/ERF74* exhibited significant high transcript levels in the S2 stage and showed decreased expression levels during flower development (Figure 5B). Also, *LrAP2/ERF01*, *LrAP2/ERF67*, *LrAP2/ERF45*, *LrAP2/ERF55*, and *LrAP2/ERF76* had considerably higher expression levels in the S3 stage. Finally, most *LrAP2/ERF* genes were upregulated in the S4 stage. The expression of other members in flowering stages ranges from moderate to high (Figure 5B). These results indicated that the *LrAP2/ERFs* gene was restructuring under time-dependent evolutionary dynamics in *Lycoris*.

### 2.7. Expression Patterns of LrAP2/ERFs in Response to Drought Stress and MeJA Treatment

**Figure 6:**
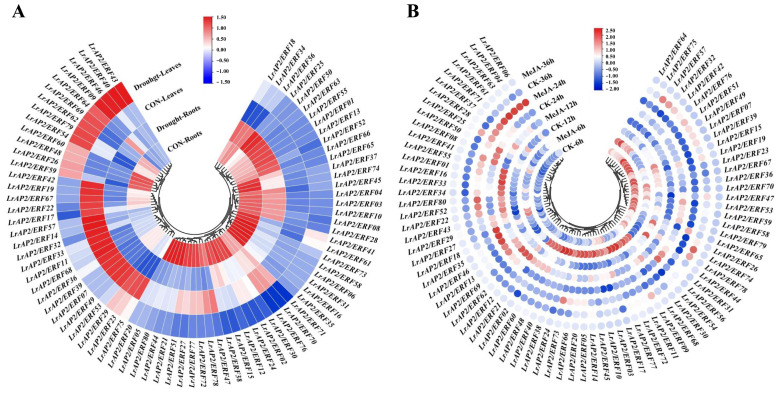
*LrAP2/ERF* gene expression profiles under drought stress and MeJA treatment. (**A**) Heatmap with hierarchical cluster analysis of drought-responsive differentially expressed *LrAP2/ERF* genes. (**B**) Heatmap of *LrAP2/ERF* gene expression profiles with MeJA treatment. Red and blue represent high and low relative transcript abundance, respectively.

We analyzed the expression patterns of drought-responsive *LrAP2/ERF* genes in *Lycoris* leaves and roots. As shown in Figure 6A, the responses of *LrAP2/ERFs* were different between individuals after drought treatment. Following 24 h of drought exposure, most *LrAP2/ERF* genes showed decreased levels in the leaves and roots of *L. radiata*, although the expression patterns of these genes were statistically significantly greater than the control group (Figure 6A). However, the majority of *LrAP2/ERF* genes in *L. radiata* roots progressively decreased after being subjected to drought conditions. Moreover, the expression of *LrAP2/ERF01*, *LrAP2/ERF13*, *LrAP2/ERF25*, *LrAP2/ERF50*, *LrAP2/ERF52*, *LrAP2/ERF55*, *LrAP2/ERF63*, and *LrAP2/ERF66* reached the highest level under drought stress in *L. radiata* roots. In *L. radiata* leaves, 13 *LrAP2/ERF* genes (*LrAP2/ERF09*, *LrAP2/ERF26*, *LrAP2/ERF40*, *LrAP2/ERF43*, *LrAP2/ERF46*, *LrAP2/ERF48*, *LrAP2/ERF54*, *LrAP2/ERF59*, *LrAP2/ERF60*, *LrAP2/ERF62*, *LrAP2/ERF64*, *LrAP2/ERF69*, and *LrAP2/ERF79*) were significantly up-regulated under drought treatment. Also, the expression levels of *LrAP2/ERF07*, *LrAP2/ERF11*, *LrAP2/ERF14*, *LrAP2/ERF17*, *LrAP2/ERF19*, *LrAP2/ERF22*, *LrAP2/ERF23*, *LrAP2/ERF29*, *LrAP2/ERF32*, *LrAP2/ERF33*, *LrAP2/ERF36*, *LrAP2/ERF39*, *LrAP2/ERF42*, *LrAP2/ERF49*, *LrAP2/ERF53*, *LrAP2/ERF57*, *LrAP2/ERF67*, *LrAP2/ERF68*, and *LrAP2/ERF75* were steadily reduced in leaves upon exposure to drought (Figure 6A). From these findings, *LrAP/ERF* genes are responsible for controlling physiological processes in plants.

Earlier research involving the sequencing of the *Lycoris aurea* transcriptome has shown that MeJA treatment may induce *LaAP2/ERF* gene expression [52]. Therefore, the *L. aurea* transcriptomic data were also examined for *LrAP2/ERF* genes. Orthologous transcripts for all 80 *LrAP2/ERF* genes were discovered in the *L. aurea* transcriptome data (Table S1c). Among them, 63 *LrAP2/ERFs* were up-regulated, while 17 *LrAP2/ERF* genes were suppressed with MeJA treatment for 6 h (Figure S2). Further, qRT-PCR was used to determine the expression patterns of *LrAP2/ERFs* in the *L. radiata* leaves. After being exposed to MeJA, the expression values of the majority of *LrAP2/ERFs* changed with time (Figure 6B). Following 6 h, the expression level of *LrAP2/ERFs* increased, then decreased after 12 h. In particular, the transcriptional level of 23 *LrAP2/ERF* genes (*LrAP2/ERF02*, *LrAP2/ERF03*, *LrAP2/ERF05*, *LrAP2/ERF09*, *LrAP2/ERF10*, *LrAP2/ERF11*, *LrAP2/ERF14*, *LrAP2/ERF17*, *LrAP2/ERF21*, *LrAP2/ERF20*, *LrAP2/ERF24*, *LrAP2/ERF30*, *LrAP2/ERF38*, *LrAP2/ERF40*, *LrAP2/ERF48*, *LrAP2/ERF45*, *LrAP2/ERF54*, *LrAP2/ERF60*, *LrAP2/ERF66*, *LrAP2/ERF68*, *LrAP2/ERF72*, *LrAP2/ERF73*, and *LrAP2/ERF77*) increased dramatically under MeJA treatment at 6 h, whereas the levels of the other 8 *LrAP2/ERF* genes (*LrAP2/ERF01*, *LrAP2/ERF12*, *LrAP2/ERF16*, *LrAP2/ERF33*, *LrAP2/ERF34*, *LrAP2/ERF55*, *LrAP2/ERF62*, and *LrAP2/ERF69*) increased after 12 h in the presence of MeJA compared to control treatment. Moreover, at some points in time, the expression levels of certain genes shifted substantially. Specifically, the expression levels of *LrAP2/ERF04*, *LrAP2/ERF06*, *LrAP2/ERF61*, *LrAP2/ERF63*, and *LrAP2/ERF71* at 24 h were increased relative to the control levels but gradually declined at 36 h. Additionally, the expression patterns of *LrAP2/ERF05*, *LrAP2/ERF14*, *LrAP2/ERF20*, *LrAP2/ERF48*, *LrAP2/ERF54*, *LrAP2/ERF72*, and *LrAP2/ERF77* were in line with the findings of transcriptomic analysis [52]. However, qRT-PCR analysis and transcriptomic data also showed some discordance, proving that there were variations between *L. aurea* and *L. radiata* samples. Notably, the expression performance of *LrAP2/ERF11*, *LrAP2/ERF72*, and *LrAP2/ERF77* upregulated significantly at 6 h. *LrAP2/ERF04*, *LrAP2/ERF06*, *LrAP2/ERF61*, and *LrAP2/ERF63* all showed substantial elevations in expression levels after being treated with MeJA for 24 h. Based on these findings, one might hypothesize that MeJA is responsible for regulating the expression profile of *LrAP/ERF* genes. According to these results, *LrAP2/ERFs* exhibited varied expression profiles when exposed to MeJA hormone therapy and may contribute to developmental processes through hormone signaling pathways in *Lycoris*.

### 2.8. Detection of the Primary Anthocyanin Pigments in the Petals of Lycoris

**Figure 7:**
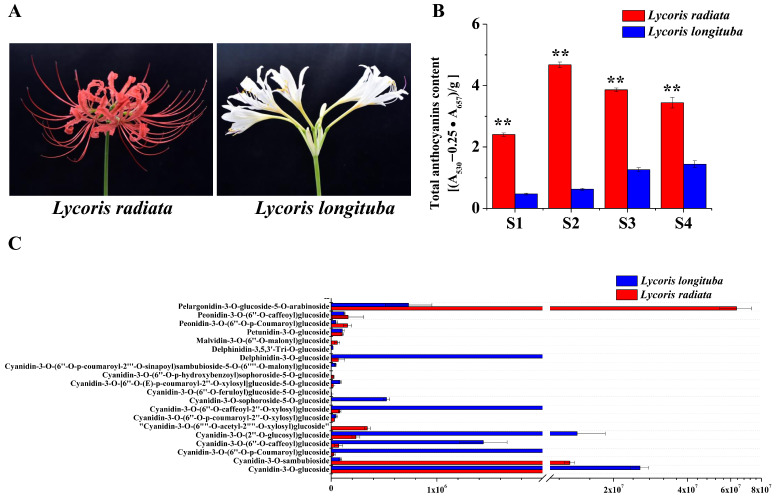
Phenotypes and anthocyanin content in petals of *L. radiata* and *L. longituba*. (**A**) Petals of *L. radiata* and *L. longituba* at flower developmental stages. (**B**) Anthocyanin levels in *L. radiata* and *L. longituba* petals at four flower developmental stages. All data were shown as mean ± SD (n = 3). Bars with different letters are significantly different at *p* < 0.01 (**). (**C**) Anthocyanins were identified and quantified from petals of *L. radiata* and *L. longituba*.

The total anthocyanins of *L. radiata* and *L. longituba* petals were determined to reveal the metabolic mechanism behind the color phenotype in these two species (Figure 7A,B). In addition, we examined how the contents of anthocyanin changed throughout the four distinct phases of petal formation in both the *L. radiata* and *L. longituba* species. Based on the findings, the total anthocyanin concentration of the *L. radiata* samples obtained from the S2 stage was remarkably increased relative to that of the other samples. The total anthocyanin content of the *L. longituba* petals was significantly lower than that of the *L. radiata* petals. These findings were in line with the degree to which the *Lycoris* petals displayed their color. To further analyze the differences in anthocyanin metabolites in the *Lycoris* petals of different colors, the anthocyanin compounds were detected by UHPLC-ESI-MS/MS. A total of 20 anthocyanins were identified and quantified from the *L. radiata* petals and *L. longituba* petals, and the relative content of all 20 anthocyanins was analyzed (Figure 7C). By comparing these anthocyanins, we observed that the *L. radiata* petals had the greatest concentration of pelargonidin-3-O-glucoside-5-O-arabinoside, followed by cyanidin-3-O-sambubioside (Figure 7C). The petals of *L. longituba* had the greatest concentrations of delphinidin-3-O-glucoside and cyanidin-3-O-(2″-O-glucosyl) glucoside. We hypothesize that the anthocyanins pelargonidin-3-O-glucoside-5-O-arabinoside and cyanidin-3-O-sambubioside were the primary compounds accountable for the red color of the petal margins in *Lycoris*.

### 2.9. Integrated LrAP2/ERF Genes and Metabolites in the Anthocyanin Biosynthesis Pathway

**Figure 8:**
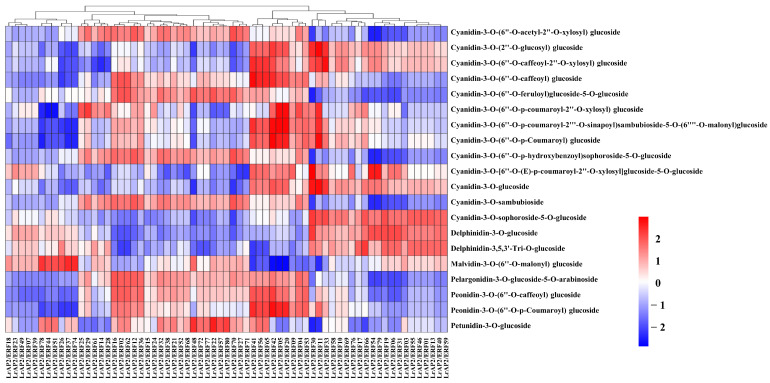
The Pearson’s correlation coefficients of *LrAP2/ERFs* with anthocyanin biosynthesis metabolites. Higher values are represented by red, whereas lower values are represented by blue.

As crucial modulators of anthocyanin accumulation, AP2/ERF TFs typically function by regulating the expression of structural genes throughout the anthocyanin biosynthetic pathways. We constructed a co-expression network of *LrAP2/ERFs* and the anthocyanin metabolic byproducts in *Lycoris* to probe into the nature of the connection between *LrAP2/ERFs* and the process of anthocyanin accumulation (Figure 8, Table S2). Calculations were made to determine the PCC between the anthocyanin metabolites and the performance of the *LrAP2/ERF* genes expression. The findings implied that nine *LrAP2/ERFs* (*LrAP2/ERF04*, *LrAP2/ERF05*, *LrAP2/ERF09*, *LrAP2/ERF20*, *LrAP2/ERF41*, *LrAP2/ERF42*, *LrAP2/ERF53*, *LrAP2/ERF56*, and *LrAP2/ERF65*) positively regulated the anthocyanin metabolites, whereas 11 *LrAP2/ERFs* (*LrAP2/ERF74*, *LrAP2/ERF37*, *LrAP2/ERF26*, *LrAP2/ERF51*, *LrAP2/ERF44*, *LrAP2/ERF78*, *LrAP2/ERF39*, *LrAP2/ERF07*, *LrAP2/ERF49*, *LrAP2/ERF23*, and *LrAP2/ERF18*) inversely controlled anthocyanin metabolic. In addition, pelargonidin-3-O-glucoside-5-O-arabinoside was found to have a significant correlation with the expression of *LrAP2/ERF* TFs, demonstrating that pelargonidin-3-O-glucoside-5-O-arabinoside performs an instrumental function in the red coloring of petals in *L. radiata*. The expression level of *LrAP2/ERF16* was shown to have a notable positive link to the amount of pelargonidin-3-O-glucoside-5-O-arabinoside present in the samples (Figure 8 and Table S2), indicating that *LrAP2/ERF16* may play a crucial part in the accumulation of pelargonidin.

**Figure 9:**
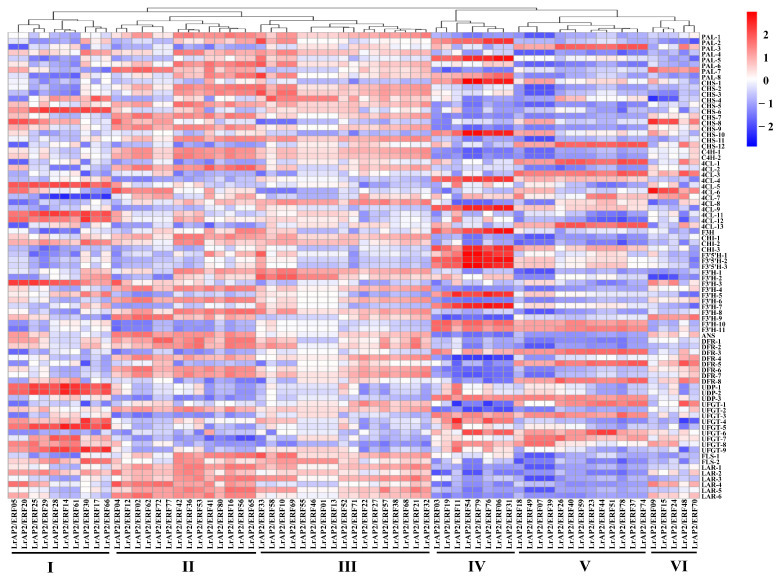
The Pearson’s correlation coefficients of *LrAP2/ERFs* with anthocyanin biosynthesis DEGs. The enzymes include 4-coumarateCoA ligase (*4CL*), phenylalanine ammonia lyase (*PAL*), chalcone synthase (*CHS*), flavone 3-hydroxylase (*F3H*), chalcone isomerase (*CHI*), flavonoid 3′-hydroxylase (*F3′H*), dihydroflavonol reductase (*DFR*), flavonol synthase (*FLS*), UDP-flavonoid glucosyl transferase (*UFGT*), anthocyanidin reductase (*ANR*), and leucoanthocyanidin reductase (*LAR*). Higher values are represented by red, whereas lower values are represented by blue.

**Figure 10:**
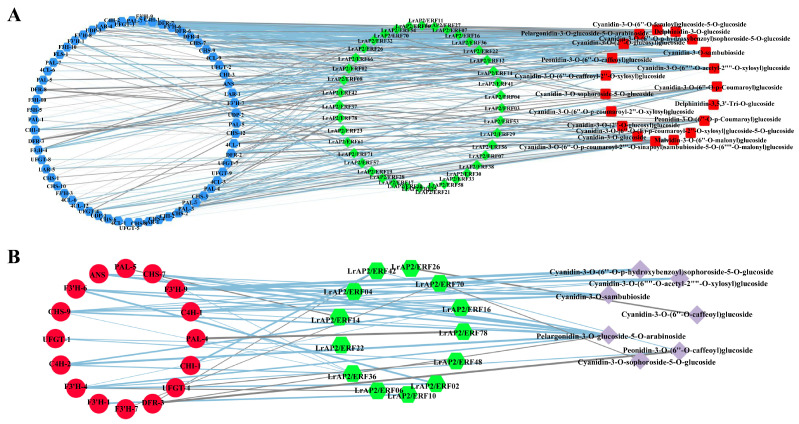
The regulatory network of key anthocyanin biosynthesis genes, metabolites and *LrAP2/ERF* genes in *Lycoris*. (**A**) Correlation network of *LrAP2/ERF* genes and anthocyanin biosynthesis genes involved in anthocyanin biosynthetic pathways. r represents the Pearson correlation coefficient; relation represents the correlation, including positive (r > 0.8) and negative correlations (r < −0.8). (**B**) Correlation network of metabolite-related *LrAP2/ERF* genes and anthocyanin biosynthesis DEGs involved in anthocyanin biosynthetic pathways. r represents the Pearson correlation coefficient; relation represents the correlation, including positive (r > 0.9) and negative correlations (r < −0.9).

According to the findings of the co-expression study, the relationship between the *LrAP2/ERF* genes and the anthocyanin biosynthesis genes in *Lycoris* may be clustered into six primary clusters (Figure 9 and Table S3). Multiple *LrAP2/ERFs* genes in cluster II (*LrAP2/ERF16*, *LrAP2/ERF36*, *LrAP2/ERF41*, *LrAP2/ERF42*, *LrAP2/ERF53*, *LrAP2/ERF56*, *LrAP2/ERF65*, and *LrAP2/ERF80*) displayed positive correlations with structural genes in the anthocyanin pathway. Conversely, *LrAP2/ERFs* in cluster V (*LrAP2/ERF07*, *LrAP2/ERF18*, *LrAP2/ERF39*, and *LrAP2/ERF49*) demonstrated an inverse link to anthocyanin biosynthesis pathway genes in *Lycoris*. Some *LrAP2/ERFs* genes in cluster IV (*LrAP2/ERF16*, *LrAP2/ERF36*, *LrAP2/ERF41*, *LrAP2/ERF42*, *LrAP2/ERF53*, *LrAP2/ERF56*, *LrAP2/ERF65*, and *LrAP2/ERF80*) had a considerable positive correlation with *PAL-2*, *PAL-4*, *CHS-10*, *4CL-4*, *4CL-10*, *F3′5′H-1*, *F3′5′H-2*, *F3′5′H-3*, *F3′H-5*, and *F3′H-7* genes in the anthocyanin pathway. Cluster II *LrAP2/ERFs* were co-expressed with genes involved in the anthocyanin pathway, and *LrAP2/ERF02*, *LrAP2/ERF04*, *LrAP2/ERF16*, *LrAP2/ERF36*, and *LrAP2/ERF42* were chosen for their ability to regulate the accumulation of anthocyanins. It was observed that the identified anthocyanin biosynthesis genes and key transcription factors were remarkably linked to seven anthocyanins: cyanidin-3-O-sambubioside, peonidin-3-O-(6″-O-caffeoyl)glucoside, cyanidin-3-O-sophoroside-5-O-glucoside, cyanidin-3-O-(6″-O-p-hydroxybenzoyl) sophoroside-5-O-glucoside, cyanidin-3-O-(6″-O-acetyl-2″-O-xylosyl)glucoside, cyanidin-3-O-(6″-O-caffeoyl) glucoside, and pelargonidin-3-O-glucoside-5-O-arabinoside (Figure 10, Tables S2 and S3). The aggregation of nine anthocyanins and anthocyanin enzyme-encoding genes was favorably correlated with the expression of several key *LrAP2/ERFs* (*LrAP2/ERF02*, *LrAP2/ERF10*, *LrAP2/ERF16*, and *LrAP2/ERF36*), suggesting that they might be involved in anthocyanin accumulation and contribute to the red color of *L. radiata* petals.

### 2.10. PPI Networks of LrAP2/ERF Proteins

**Figure 11:**
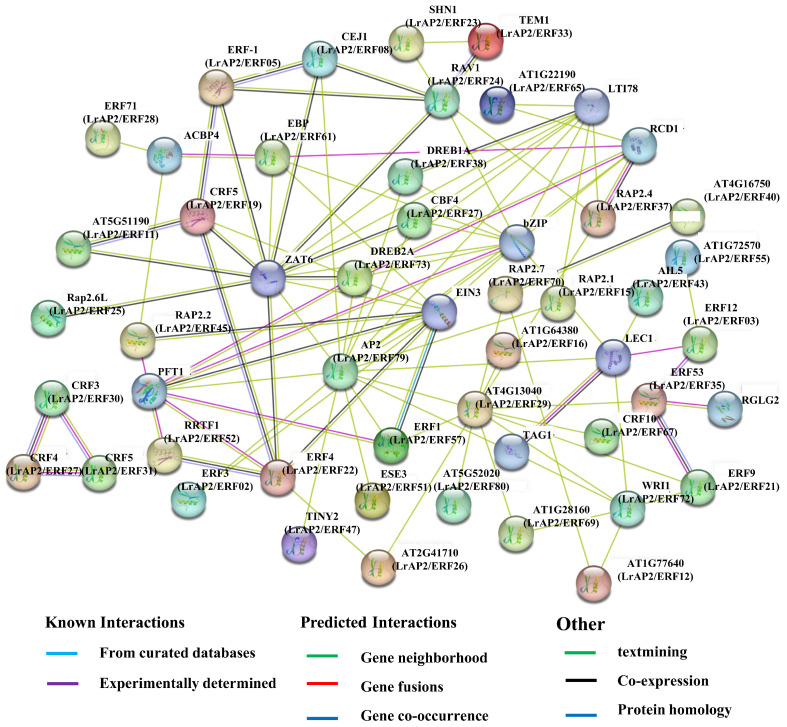
The predicted network of protein-protein interactions between LrAP2/ERFs by STRING database. Different colors represent different interaction types. *Arabidopsis* AP2/ERF names are marked, whereas their homologs in *Lycoris* are in parentheses.

Many LrAP2/ERF proteins are predicted to interact with each other by the STRING database depending on their shared *Arabidopsis* orthologs (Tables S4 and S5, Figure 11), in agreement with previous studies indicating that the binding activity of AP2/ERF proteins depends on their assembly into homodimers or heterodimers [6]. We hypothesized the existence of multiple crucial interactions (Figure 11). A PPI network including AP2/ERF was built using the freely available STRING 11.5 program to detect interacting proteins. According to the findings, these five proteins: AP2 (LrAP2/ERF79), ERF4 (LrAP2/ERF22), DREB2A (LrAP2/ERF23), DREB1A (LrAP2/ERF38), CRF4 (LrAP2/ERF19), and ERF4 (LrAP2/ERF22), perform a pivotal function in a complex regulatory network by interacting with other TFs. Key proteins were chosen to subsequently investigate the regulatory network governing floral growth and abiotic stresses (Figure 11). Only a small number of AP2/ERF TFs have been involved in the control of flowering time in *Arabidopsis* [29]. In this study, LrAP2/ERF79 was orthologous to ERF2 (Table S5); hence, *LrAP2/ERF79* could also have a crucial function in *Lycoris* flower formation. *ERF4* contributed to the synthesis of anthocyanins in response to illumination in *A. thaliana*. Meanwhile, *LrAP2/ERF22*, specifically expressed in roots, was considered a key factor involved in drought stress and anthocyanin metabolism via the mechanism of responding to phytohormones because its ortholog *ERF4* in *A. thaliana* has been shown to play similar functions. This network consists of proteins analogous to CBF3 (LrAP2/ERF30, LrAP2/ERF32, and LrAP2/ERF42), one that is similar to CBF4 (LrAP2/ERF27), and one that is similar to CBF5 (LrAP2/ERF31). *CBF3*, *CBF4*, and *CBF5* were important TFs in the ICE1-CBF-COR signaling pathway, which meant they were involved in more robust abiotic-response networks because CBF3, CBF4, and CBF5 were the key TFs in the core mechanism of abiotic stress mediated by the ICE1-CBF-COR signaling pathway. Furthermore, *CBF4* was involved in the response to drought stress but not to low temperatures; LrAP2/ERF27 and CBF4 might have comparable functions. LrAP2/ERF38 and LrAP2/ERF73 have a lot of similarities with DREB1A and DREB2A in terms of their protein sequences, highlighting their indispensable functions in adaptations to drought. The results largely confirm the predicted interaction networks and imply that they may exert similar functions in *Lycoris*.

## 3. Discussion

### 3.1. Characterization of LrAP2/ERF Genes in Lycoris

In plants, the AP2/ERF transcription factor family, an essential regulator in many biological processes, plays vital roles in diverse processes, including plant growth, fruit ripening and softening, flower development, and biotic and abiotic stress responses. Recently, many *AP2/ERF* genes have been confirmed in different species, but the number of *AP2/ERF* genes varies greatly. For example, 147 *AP2/ERF* genes in *A. thaliana* [2], 165 *AP2/ERF* genes in *Oryza sativa* [15], 120 *AP2/ERF* genes in Rhododendron (*Rhododendron simsii*) [23], 229 *AP2/ERF* genes in *Zea mays* [24], and 234 *AP2/ERF* genes in *Pyrus pyrifolia* [25] were identified. Although the *AP2/ERF* TF family has been found in several plant species, very little is known about its involvement in *Lycoris*. Using recently reported transcriptomic data, we performed a homolog search and domain analysis, ultimately identifying 80 *LrAP2/ERF* genes (Table S1). In comparison to *AP2/ERFs* in *A. thaliana* and other plants, the *LrAP2/ERFs* shared traits such as the amount of encoding amino acids, motifs, and isoelectric point [2]. To categorize the AP2/ERF groups in *L. radiata*, phylogenetic analysis, full-length protein sequences, and the classification of AP2/ERF groups in *Arabidopsis* [2] were integrated into this study.

Among transcription factors, conserved motifs play a significant role in gene functioning. Motif analysis of *LrAP2/ERF* genes on the same branch commonly has a similar domain component. These results can be used as an important basis for AP2/ERF protein classification (Figure 2). Target gene transcription activities and PPI are linked to these motifs [6]. Consequently, conserved motif analysis may be used to evaluate the characterizations and roles of LrAP2/ERF TFs. All of the LrAP2/ERFs contained over two preserved motifs, which agrees with past findings from other plant species [15,16,17,18,19,20,21,22,23,24,25,26]. Each group member had the same or similar motif, suggesting that AP2/ERF TFs in the same group have similar protein structures and biological functions. Transcription factors always harbor some important conserved domains and motifs for their regulatory function. In *Lycoris*, Motif 1, Motif 2, and Motif 7 are typically the most conserved regions of the AP2/ERF proteins, and all LrAP2/ERFs contained at least one of these three motifs, indicating that the AP2 domain is also highly conserved in LrAP2/ERFs. Besides the AP2 domain-related motifs, the other motifs are present in subfamily-specific distributions. These specific motifs in different subfamilies may have important functions. Thus, these results show that although most motifs in the AP2/ERF proteins were strongly conserved, some specific motifs could be associated with novel functions in *Lycoris*, which demands in-depth investigation.

### 3.2. The AP2/ERF Gene Expression Patterns Facilitate Their Functional Analysis

We methodically analyzed the expression patterns of *LrAP2/ERF* TFs in various tissues to investigate their physiological role in *Lycoris* biological processes. Homologous genes with similar expression profiles are conserved due to the dosage effect, whereas homologous genes with different expression profiles are retained by functionalization and subfunctionalization [6]. Previous reports have demonstrated that AP2/ERF family members are widely expressed in various tissues and organs, regulating plant growth and development [23,25]. We also performed the transcriptomic and qRT-PCR analyses from different tissues of two species of *Lycoris* to determine the *AP2/ERF* gene expression (Figure 4). *LrAP2/ERF* genes were expressed in at least one tissue, and their expression patterns varied markedly among the different tissues. These tissue-specific expression patterns propose that *LrAP2/ERFs* may be involved in *Lycoris* tissue-specific developmental and signal transduction processes.

### 3.3. AP2/ERFs Are Associated with Lycoris Flower Development

As an important part of plant life history, flowering plays a great role in plant reproduction and survival [26,27,28,29,30]. Several studies have shown that the *AP2/ERF* genes are involved in flower development. For instance, *CsERF110* has been related to ethylene signaling and floral sex differentiation in cucumber [53]. *ERF110* was discovered to affect the flowering of *Chrysanthemum morifolium* and *A. thaliana* [54]. Overexpression of soybean *GmAP2* genes led to early flowering and increased the length, width, and area of seeds in *Arabidopsis* [55]. In rice, *RAV* genes are responsible for controlling the heading date as well as the development of the gynoecium [56]. The use of exogenous ethylene can potentially cause *C. moschata* to produce more female flowers [57]. In addition, the *AP2/ERF* TF gene known as late flowering semi-dwarf (*LFS*) in rice is responsible for the late flowering as well as the semi-dwarf phenotypes [27]. *AeAP2/ERF061* and *AeAP2/ERF067* are proven to play a crucial role in regulating the flowering process and the formation of floral tissues [26]. However, few studies were carried out on the molecular mechanism of flowering biology in *Lycoris,* especially for the *AP2/ERF* genes. Considering *Lycoris* is an important groundcover and ornamental flower plant widely applied in many fields, we checked *AP2/ERF* gene expression by using RNA-seq data and the qRT-PCR method in *Lycoris* so as to intuitively interpret the potential biological functions of the *AP2/ERF* gene in flowering. Over half of the *LrAP2/ERF* genes were lower expressed at the S1 stage and then markedly increased at the S4 stage. Additionally, the expression levels of *LrAP2/ERF02*, *LrAP2/ERF07*, *LrAP2/ERF48*, and *LrAP2/ERF60* in the flower S4 stages were significantly higher than other flower stages in *Lycoris*. The results confirmed that some of the *AP2/ERF* genes may play important roles in flower development.

### 3.4. AP2/ERF Family Genes Play Important Roles in Lycoris Stress Responses

Drought is one of the most serious abiotic stresses that could adversely hinder plant growth, development, and productivity. Drought stress usually causes a water deficit in plants, which is embodied in decreased height, leaf wilting, and the number and area of leaves [58]. Recent research has revealed that several genes belonging to the *AP2/ERF* family are closely connected to the effects of drought [59,60]. *ERFs* may alter the expression of genes involved in osmolyte production to impart drought resistance [6]. For example, *MdERF38* was shown to enhance the manufacture of anthocyanins in apples when exposed to drought conditions [44]. In rice, *OsERF115*/*AP2EREBP110* enhanced drought tolerance via the mechanism of elevating the expression level of the proline biosynthesis *P5CS1* gene [61]. *GmDREB1* could confer drought tolerance in soybeans by increasing the photosynthetic efficiency, the accumulation of osmoregulation substances, and the synthesis of melatonin [62]. In this investigation, we discovered a progressively elevated expression trend of *LrAP2/ERF* genes during drought stress conditions, showing that they may be extensively connected to the modulation of *L. radiata* under drought conditions (Figure 6A). A few *LrAP2/ERF* genes were significantly up-regulated under drought treatment in *L. radiata* roots and leaves. In particular, the expression level of *LrAP2/ERF64* was more fold-up-regulated, playing an important function in *L. radiata* responding to drought stress. The results of this study will lay a foundation for analyzing the drought resistance function of the *LrAP2/ERF* gene family in *L. radiata*.

Jasmonic acid is a signaling molecule that is essential for plant development and stress responses. The AP2/ERF TF family contributes significantly to the development of plants, and the activity of its members might be influenced by the presence of auxin, abscisic acid, jasmonate, or ethylene. These genes are involved in the modulation of many plant processes, like responses to stress and defense mechanisms [63]. For instance, *ERF1* responses to jasmonic acid and ethylene are known to trigger defensive mechanisms that rely on ethylene/jasmonic acid [64]. *JERF1* performs the role of a TF in various signal-transduction pathways in tobacco. It reacts to signals from ethylene, jasmonic acid, and abscisic acid by binding to the GCC box or the dehydration-responsive element (*DRE*) sequence [65]. *AtERF2* shows a positive response to the jasmonate signal-transduction cascade [66]. In soybean, the jasmonic acid and ethylene treatments induce the expression of *GmERF3*, which has an important role in responses to biotic and abiotic stresses [67]. The interplay between *MdERF3* and *MdMYC2* results in the activation of the *MdACS1* transcription, consequently performing a notable function in the ripening of apple fruits mediated by ethylene and jasmonate [68]. Furthermore, the regulation of genes associated with defense responses occurs through the synergistic regulation of *BrERF72* expression by jasmonate and ethylene [69]. We also found that 23 *LrAP2/ERF* gene transcription levels were increased rapidly under MeJA treatment at 6 h, while the other eight *LrAP2/ERF* genes were higher after 12 h of MeJA treatment (Figure 6B and Figure S2). Our findings provide an insight into the potential functional roles of the *LrAP2/ERF* family in the jasmonate signaling cascade in *Lycoris* plants.

### 3.5. LrAP2/ERFs Related to L. radiata Petal Color Change

Anthocyanins are the primary metabolites responsible for the development of petal color in plants and possess anti-inflammatory, anti-aging, and antioxidant properties. The anthocyanin biosynthesis pathway has been the subject of extensive research in a variety of plant species [70,71,72]. Existing literature demonstrates that anthocyanins responsible for regulating petal color development depend on pigmentation and the relative expression of biosynthetic genes [73,74]. Notably, the changes in the coloration of petals are principally attributed to six anthocyanidins (petunidin, peonidin, pelargonidin, malvidin, delphinidin, and cyanidin) [75,76]. In this work, a total of 20 differential anthocyanins were identified in the *L. radiata* and *L. longituba* petals and primarily contained two categories of cyaniding and pelargonidin (Figure 7). Practically, all chemicals exhibited considerably high expression in the red petals, with pelargonidin (pelargonidin-3-O-glucoside-5-O-arabinoside) perhaps serving as the primary contributor to this red coloration. Earlier academic investigations have also recognized outcomes that are comparable to our findings [76,77,78]. Additionally, certain critical structural genes involved in anthocyanin biosynthesis were also detected based on RNA-seq profiling, and they were shown to be considerably upregulated, which implied that AP2/ERF TFs exerted a stronger influence on anthocyanin [50].

Numerous studies have revealed evidence that the conservation of regulatory mechanisms in anthocyanin biosynthesis is *AP2/ERF*-based. It has been shown that several *AP2/ERFs* are necessary for the production of anthocyanin in a variety of species. Notably, the red coloration in pear fruits is co-regulated by the interaction between *PpERF3*, *PpMYB114*, and *PpbHLH3* [35]. Additionally, the interaction between *Pp4ERF24*, *Pp12ERF96*, and *PpMYB114* is responsible for modulating the biosynthesis of anthocyanin induced by blue light in ‘Red Zaosu’ pear fruit, thus boosting *PpUFGT* expression [43]. Also, the *MdERF1B* regulator is shown to interface with both *MdMYB9* and *MdMYB11* in addition to binding to their promoters, thus promoting the accumulation of proanthocyanin and anthocyanin [42]. *MdERF38* interacts with *MdMYB1* to stimulate the production of anthocyanin when exposed to drought conditions. *MdERF38* is susceptible to posttranslational degradation by *MdBT2* [44]. *FaRAV1* exerts a positive modulation of the accumulation of anthocyanin in strawberries by activating genes in the anthocyanin pathway and increasing the levels of *FaMYB10* [78]. In the early phases of apple color development, the MdERF109 protein is responsible for the induction of the expression of anthocyanin-associated genes as well as the aggregation of anthocyanins [45]. *ERF5* can control the anthocyanin production process in mulberry species as it interacts with *F3H* and *MYBA* genes [39]. Nonetheless, it is still unknown whether *LrAP2/ERFs* participate in anthocyanin biosynthesis in *Lycoris*. In our research, *LrAP2/ERF3* showed significantly different expression levels in petal, which might be candidate regulators of anthocyanin accumulation in petal color changes in *L. radiata* (Figure 8, Figure 9 and Figure 10). The gene-metabolite correlation network showed that the metabolite pelargonidin-3-O-glucoside-5-O-arabinoside was strongly correlated with *LrAP2/ERF16* in anthocyanin biosynthesis pathways (Figure 10). Interestingly, our results also showed that the *LrAP2/ERF16* transcription factor specifically interacted with anthocyanin biosynthesis structure genes, suggesting that it might be involved in petal coloration in *Lycoris* (Figure 8). Therefore, more research is needed to understand how *AP2/ERF* has evolved throughout angiosperms and what function it serves in other lineages. These results further verified the roles of key genes and metabolites involved in anthocyanin accumulation during petal color changes.

## 4. Materials and Methods

### 4.1. Transcriptome-Wide Identification and Expression Profiling of Lycoris AP2/ERFs

The transcript database of *L. radiata* flowers was searched for RNA-Seq data containing 87,584 unigenes [50]. Among them, 147 AtAP2/ERF proteins retrieved from TAIR (Arabidopsis information resource database, https://www.arabidopsis.org/, accessed on 5 June 2022) were used in the assessment of the sequence homology with *L. radiata* transcriptomes from the database by basic local alignment (BLASTn) [19]. In all, 80 genes in *Lycoris* that belong to the AP2/ERF family (designated as *LrAP2/ERF1*-*LrAP2/ERF80*) were discovered through gene annotation and bioinformatics analysis. To screen for *LrAP2/ERF* genes from *L. radiata*, we employed the hidden Markov model (HMM) profile of the AP2/ERF domain (protein family ID: PF00010) derived from the Pfam protein family database (http://pfam.xfam.org/, accessed on 5 June 2022). Then, we applied the default settings in the NCBI BatchWeb CD-Search Tool (https://www.ncbi.nlm.nih.gov/Structure/bwrpsb/bwrpsb.cgi, accessed on 5 June 2022) to verify the AP2/ERF domain in the putative *LrAP2/ERF* TFs. A thorough investigation illustrated that this characteristic has a high-confidence association with the conserved domain. The sequences that were expected to be specific hits were kept for subsequent investigation (Table S1). Additionally, to ascertain whether or not each candidate protein sequence had an AP2/ERF domain, we consulted the PFAM and SMART (http://smart.embl-heidelberg.de/, accessed on 7 June 2022) databases. Finally, we leveraged the ExPASy online platform to figure out the whole length of the amino acid sequences, as well as their protein instability index, molecular weights (MWs), and isoelectric points (PIs) [79].

### 4.2. Phylogenetic Tree and Protein Motif Analyses of LrAP2/ERF Proteins

To execute a phylogenetic analysis, the AtAP2/ERF amino acid sequences were extracted from the TAIR database. By applying the neighbor-joining approach (https://www.megasoftware.net/, accessed on 7 June 2022), the phylogenetic tree of AP2/ERFs from *A. thaliana* and Lycoris was designed utilizing MEGA7. After that, LrAP2/ERF proteins were categorized according to the evolutionary connections that they shared with AtAP2/ERF proteins. Ten conserved motifs in the LrAP2/ERF superfamily were screened from MEME (Multiple EM for Motif Elicitation, version 5.1.1) (http://meme-suite.org/tools/meme, accessed on 7 June 2022), with each gene having motif numbers within the range of 6–50 for each gene. The SMART tool (http://smart.embl.de/, accessed on 7 June 2022) was adopted in the search for motifs throughout protein databases.

### 4.3. GO and KEGG Annotation of LrAP2/ERFs

KOBAS was utilized for Gene Ontology (GO) functional annotation [80]. GO may be classified into three different ontologies, including molecular functions (MFs), cellular components (CCs), and biological processes (BPs). A lower *p*-value was utilized to ascertain the significance of the terms. KASS was utilized for KEGG annotations [81].

### 4.4. Plant Materials and Plant Treatments

The plant materials, including *L. longituba* and *L. radiata,* were collected from Nanjing Botanical Garden, Mem. Sun Yat-Sen (118°83′ E, 32°05′ N). Nanjing, China. Specimens (no. NAS0626770 and no. NAS0626728) were stored at the herbarium of the Institute of Botany, Jiangsu Province, and the Chinese Academy of Science. Collection information and characteristics of *L. longituba* and *L. radiata* are provided in Table S6. The voucher specimens were identified by Prof. Feng Peng and deposited in the herbarium of the Institute of Botany, Jiangsu Province, and the Chinese Academy of Sciences. The plant materials used in this study were cultivated in the nursery of Nanjing Botanical Garden, Mem. Sun Yat-Sen, Nanjing, China. Seeds of *L. radiata* were collected from the nursery in November of 2021. After being treated with 75% alcohol (*v*/*v*) to sterilize them, the seeds were allowed to germinate in darkness for 2 weeks in a medium containing 1/2 MS (Murashige and Skoog, pH 5.8) at 22 °C. After that, the *L. radiata* seedlings were transferred into plastic pots with a mixture of vermiculite and soil (1:1, *v*/*v*) and maintained in a plant growth chamber under the following conditions: 16 h light/8 h dark cycle at 25 °C/22 °C, and 120 μmol m^−2^ s^−1^ irradiation). Following a year of development, the seedlings were exposed to drought stress (20% *w*/*v* polyethylene glycol (PEG6000)) for 24 h and 100 μmol L^−1^ methyl jasmonate (MeJA) for 0 h, 6 h, 12 h, 24 h, and 36 h. Three runs were completed for each treatment. The roots, flower stalks, seeds, stamens, petals, leaves, bulbs, and gynoeciums of *L. radiata* were examined to ascertain the tissue-specific transcription patterns of 80 *LrAP2/ERF* genes. Liquid nitrogen was added to promptly freeze the samples, which were then maintained at −80 °C.

### 4.5. Expression Profile Analysis and Real-Time Quantitative PCR Analysis of LrAP2/ERFs Genes

Previous research on gene expression at various phases of different tissues, floral development, and in response to MeJA exposure provided the source for the RNA-seq data used to characterize the *LrAP2/ERF* genes [50,51]. The expression profiles of the *LrAP2/ERF* genes were analyzed with the use of FPKM values. The TBtools program was adopted to establish the heatmaps for *LrAP2/ERF* expression. For RNA extraction, samples (1 g) were crushed into a fine powder with liquid nitrogen and a mortar–pestle. Then total RNA was isolated from the samples by using a RNAprep Pure Plant Kit (Cat BC508, Huayueyang, Beijing, China) and treated with RNAse-free DNAse I to eliminate residual genomic DNA. Then, agarose gel electrophoresis and spectrophotometric analysis were performed to check RNA integrity and quality. After synthesizing cDNA with the PrimeScript™ II 1st Strand cDNA Synthesis Kit (RR047A, TaKaRa Bio, Dalian, China), qRT-PCR assays were completed. Analysis of the relative levels of gene expression in 15 μL reactions was done utilizing qRT-PCR with SYBR^®^ Premix Ex Taq™ II (Tli RNaseH Plus; Takara Bio, Dalian, China) on a Bio-Rad iQ5 Real-Time PCR System (Bio-Rad, Hercules, CA, USA). For each reaction, the mixture comprised a combination of 0.6 μL of 20 μM forward and reverse primers, 4.9 μL of ddH_2_O, 2 μL cDNA, and 7.5 μL 2× TransStart^®^ Top Green qPCR SuperMix. Below are the parameters for the RT-qPCR protocol: 95 °C for 5 min; denaturation at 95 °C for 5 s; 60 °C for 30 s; 40 cycles. The specific primer pairs of the selected genes (Table S7) were designed using Primer3 (https://bioinfo.ut.ee/primer3-0.4.0/, accessed on 7 June 2022). To normalize relative levels of target gene expression via the 2^−ΔΔCt^ method [82], the *LrTIP41* gene [50] was chosen to serve as the reference gene according to a previous study on *L. aurea* [83].

### 4.6. Measurement of Total Anthocyanins

The extraction and determination of anthocyanins in *Lycoris* flowers were performed according to the method described previously [84], with minor modifications. Briefly, fresh petals (approximately 0.1 g) were ground in 1 mL of acidic methanol (0.1 mol L^−1^ HCl) and then incubated overnight in the dark at 4 °C with gentle shaking. After centrifugation at 12,000 rpm for 10 min, the supernatant was diluted four times with acidic methanol, and the absorbance was measured at 530 and 657 nm using a UV-1600 spectrophotometer (SHIMADZU, Kyoto, Japan). The concentration of anthocyanins was calculated using the following formula: Anthocyanins = (A_530_ − 0.25 × A_657_) × FW^−1^, where Anthocyanins are the amount of anthocyanins, A_530_ and A_657_ are the absorption at the indicated wavelengths, and FW represents the fresh weight of the sample.

### 4.7. Anthocyanin Quantification and Data Analysis of Lycoris

The collection of metabolite data was accomplished with the assistance of a UPLC-ESI-MS/MS system (Shim-pack UFLC SHIMADZU Nexera X2) (ultrafast liquid chromatography) instrument coupled with an MS/MS (Applied Biosystems 4500 QTRAP). At 40 °C, chromatographic isolation was performed with the aid of a Waters ACQUITY UPLC HSS T3 system (Shim-pack UFLC SHIMADZU CBM30A) that was fitted with a C18 column (250 mm × 4.6 mm × 5 μm). The amount of the injection was 5 μL, and the rate of flow was 0.4 mL/min. The formic acid concentration in the organic phase was 0.1%, while the formic acid concentration in the mobile phase was 0.1%. Below are the parameters used in the elution gradient: 0 min, 95:5 water/acetonitrile (*v*/*v*); 9.0 min, 5:95 water/acetonitrile; 10.0 min, 5:95 water/acetonitrile; 11.1 min, 95:5 water/acetonitrile; and 14.0 min, 95:5 water/acetonitrile. The metabolites eluted from the HPLC were monitored for each period by scheduled multiple reaction monitoring (MRM). The program known as Analyst 1.6.3 (Metware Biotechnology Co., Ltd., Wuhan, China) was used to perform transformations and analyses on the MRM signals. Both the commercially available Metabolites Database [85] (Metware Biotechnology Co., Ltd., Wuhan, China) and freely accessible metabolite databases [86] were used in the process of identifying and quantifying metabolites. By integrating the Pearson correlation coefficient (│PCC│ > 0.8 or │PCC│ > 0.9) between structural genes, anthocyanin metabolites, and TFs, we generated the transcriptional regulatory networks. Cytoscape was then used to provide visual representations of the regulatory network maps (v.3.7.2, USA).

### 4.8. Prediction PPI Network of LrAP2/ERF Proteins

Potential protein–protein interaction networks were predicted using the STRING.410 online database based on *A. thaliana* homologous proteins [87] (https://string-db.org, accessed on 27 June 2022). *A. thaliana* was used as the comparative organism, while the protein sequences of *LrAP2/ERFs* were uploaded to the server. After the execution of blasting with the highest bitscore, the *LrAP2/ERFs* gene interaction network was developed.

### 4.9. Statistical Analysis

Every experiment was conducted a minimum of three times separately. The results were represented as the mean ± SD of biological triplicates. In the analysis of the data, we employed the student *t*-test. * *p* < 0.05 and ** *p* < 0.01 denote the significance criterion.

## 5. Conclusions

In summary, a total of 80 *LrAP2/ERF* genes were identified from the *L. radiata* and *L. longituba* transcriptome data. These genes were then enriched by systematically analyzing basic biochemical information, phylogenetic linkages, conserved motifs, and gene expression profiles. It was discovered that the majority of *LrAP2/ERF* genes may perform numerous key functions in the growth and development of *Lycoris*, particularly in the flowering phases. The analysis of the expression pattern of *LrAP2/ERF* genes in response to drought stress and MeJA treatment suggested that the *LrAP2/ERF* genes could be significantly involved in drought stress as well as in the JA signaling pathway. An investigation into the gene-metabolite correlation network illustrated that *LrAP2/ERFs* could act as a modulator in the anthocyanin biosynthetic pathway. The functions as well as the modulatory processes in *Lycoris* were determined via a PPI analysis of *LrAP2/ERF* proteins. In conclusion, this study provides valuable information for further research on the regulatory mechanisms of *AP2/ERF* TFs in plant growth, secondary metabolism, and resistance to various stressors.

## Data Availability

All data are displayed in the manuscript.

## Supplementary Materials

The following supporting information can be downloaded at: https://www.mdpi.com/article/10.3390/ijms241914464/s1.
