# Supplementary material for: Functional Characterization of AP2/ERF Transcription Factors during Flower Development and Anthocyanin Biosynthesis Related Candidate Genes in Lycoris
Source: Int J Mol Sci. 2023 Sep 23;24(19):14464. doi: 10.3390/ijms241914464 (PMC10572147; doi:10.3390/ijms241914464)
Supplement: Supplementary file 1 [file ijms-24-14464-s001.zip › ijms-2610519-supplementary figures.pdf]

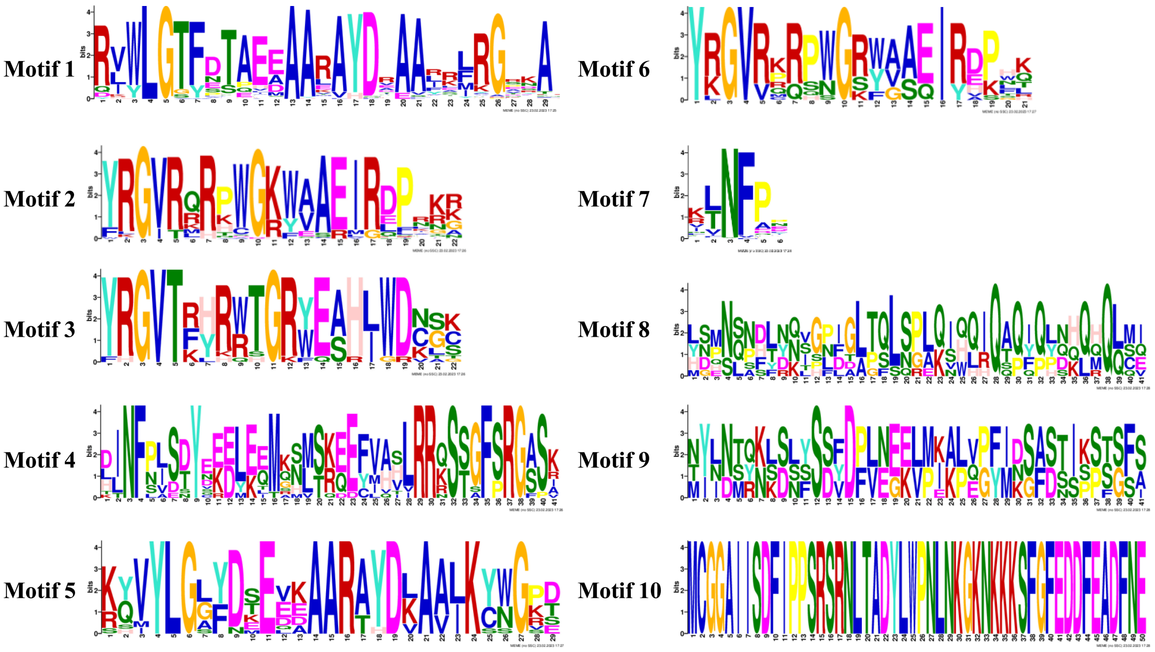

Figure S1. Conservation and diversity of the motifs in LrAP2/ERF proteins. The schematic repre-sentation of ten motifs in *AP2/ERF* family is elucidated by MEME.

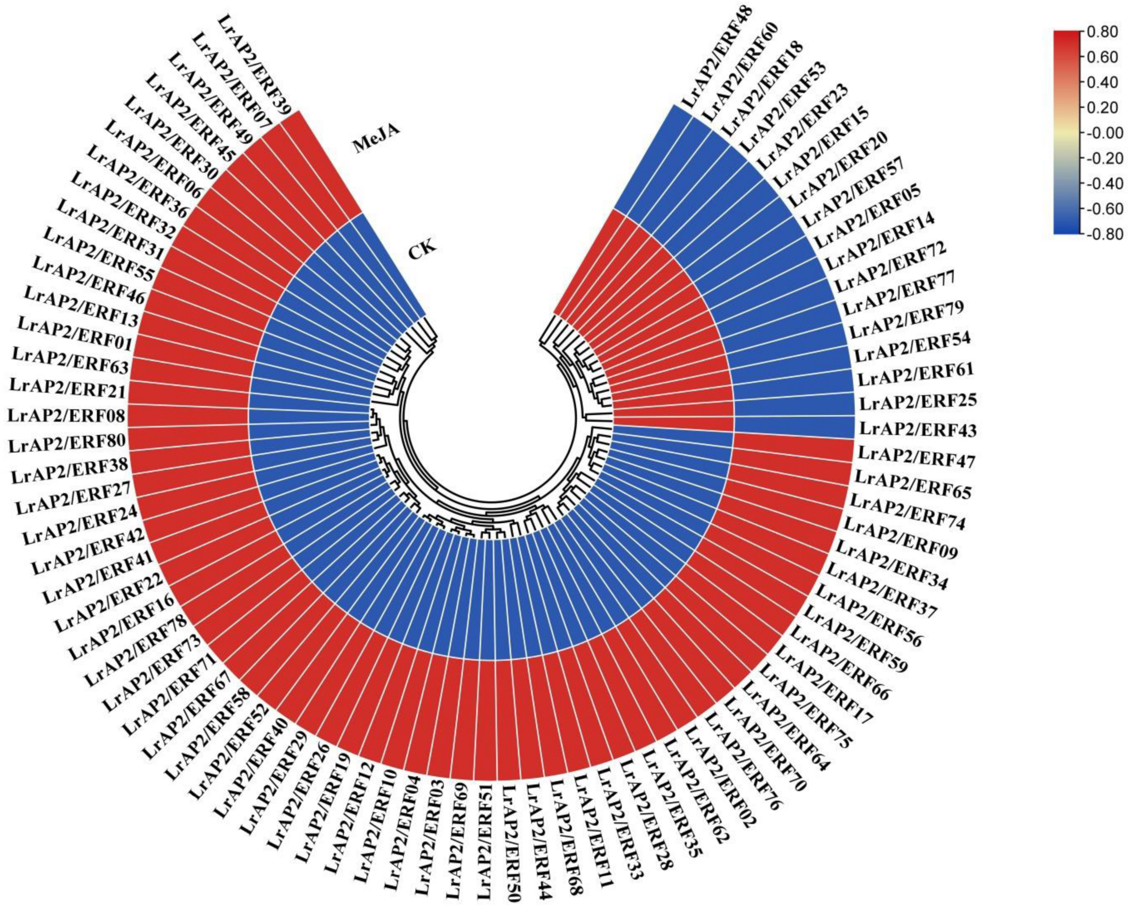

Figure S2. Heatmap of *LrAP2/ERF* genes expression profiles with MeJA treatment. Red and blue represent high and low relative transcript abundance, respectively.
